# Epigenetic Underpinnings of Inflammation: A Key to Unlock the Tumor Microenvironment in Glioblastoma

**DOI:** 10.3389/fimmu.2022.869307

**Published:** 2022-04-29

**Authors:** Nian Chen, Cheng Peng, Dan Li

**Affiliations:** State Key Laboratory of Southwestern Characteristic Chinese Medicine Resources, School of Pharmacy, Chengdu University of Traditional Chinese Medicine, Chengdu, China

**Keywords:** glioblastoma, inflammation, microenvironment, epigenetic regulation, GBM tumor cells, glioma-associated microglia/macrophages

## Abstract

Glioblastoma (GBM) is the most common malignant brain tumor in adults, and immunotherapies and genetic therapies for GBM have evolved dramatically over the past decade, but GBM therapy is still facing a dilemma due to the high recurrence rate. The inflammatory microenvironment is a general signature of tumors that accelerates epigenetic changes in GBM and helps tumors avoid immunological surveillance. GBM tumor cells and glioma-associated microglia/macrophages are the primary contributors to the inflammatory condition, meanwhile the modification of epigenetic events including DNA methylation, non-coding RNAs, and histone methylation and deacetylases involved in this pathological process of GBM, finally result in exacerbating the proliferation, invasion, and migration of GBM. On the other hand, histone deacetylase inhibitors, DNA methyltransferases inhibitors, and RNA interference could reverse the inflammatory landscapes and inhibit GBM growth and invasion. Here, we systematically review the inflammatory-associated epigenetic changes and regulations in the microenvironment of GBM, aiming to provide a comprehensive epigenetic profile underlying the recognition of inflammation in GBM.

## Introduction

Glioblastoma (GBM) is the most aggressive and common malignant brain tumor arising from neural precursor cells (1). It is defined as grade IV glioma by WHO and divided into two distinct subgroups, namely *IDH* wild-type (primary GBM, approximately 90%) and *IDH* mutant-type (secondary GBM, 10%) (2). Both types of GBM shows similar clinical symptoms that include headache, nausea, dizziness, speech difficulties, and cerebral edema (3), and is characterized by poor survival and remarkably high tumor heterogeneity, which invades surrounding brain tissue and quickly develops resistance to therapy (4). Primary GBM is more common and manifests as *de novo* development with no identifiable precursor lesion, whereas secondary GBM is characterized by precursor diffusion or progression with anaplastic astrocytoma, anaplastic oligoastrocytoma, and anaplastic oligodendroglioma (3, 5). Additionally, *EGFR* mutations are frequently enriched in primary GBM while *p53* mutations are more common in secondary GBM (6, 7). Although the differences between the two subtypes have been found, improvement of therapies for GBM still needs to continue due to unknown risk factors (8). Notably, epigenetic variations have displayed vital roles in the development of tumor progression in recent years (9), such as DNA methylation, histone modification, and other epigenetic modifiers that could modulate the oncogene expression of GBM (10, 11). Thus, epigenetic changes may be a novel perspective for understanding the physiology and pathogenesis of GBM.

The tumor microenvironment (TME) is a host for supporting the growth and invasion of tumors, promoting neoplastic transformation, protecting the tumor from host immunity, and providing niches for dormant metastases to thrive (12), and is created by interactions between malignant and non-transformed cells (13). Besides high tumor heterogeneity, a unique TME is one of vital reasons for dismal results of GBM treatment (5, 14–16). GBM tumor cells have the ability to transform the immune response into chronic inflammation (17), which helps tumor relapse by nurturing GBM stem cells (GSCs), resulting in epithelial-mesenchymal transition and multidrug resistance (18). And then, angiogenesis and inflammatory cytokines induced by GBM expansion increase the abnormal blood brain barrier (BBB) or blood tumor barrier (BTB), which further inhibits the entrance of functional immune cells in the brain (19). Apart from tumor cells, glioma-associated microglia/macrophages (GAMs) are the main types of infiltrating immune cells, and account for approximately 30% of the GBM cell population (20). Notably, GAMs acquire two specific phenotypes including the classical pro-inflammatory state M1 and alternative anti-inflammatory state M2. The latter is found to be the primary phenotype in GBM (21) and can be reprogrammed by GBM tumor cells (22), generating a complex and dynamic network of inflammatory cytokines, chemokines, and matrix remodeling (13). As a result, the autocrine and reciprocal paracrine of GBM tumor cells and GAMs promote an inflammatory TME in favor of tumor promotion (23). Moreover, inflammation cooperates with the Warburg effect in GBM to exacerbate the inflammation response in the TME through producing more inflammatory cytokines, lactate, and immunosuppressive and angiogenetic factors, leading to a more suitable and beneficial condition for GBM progression (24–32). Therefore, targeting the inflammatory TME might be a promising therapeutic strategy for GBM.

Epigenetic modifications are involved in inflammatory procession in GBM *via* regulating the inflammatory signaling pathways and production of inflammatory cytokines (24, 33–35). Due to the sustained exploration of epigenetic modification in the last decade, various epigenetic changes such as DNA methylation, histone modification, mRNA and non-coding RNA (ncRNA)-mediated targeting regulation, and nucleosome and chromosome remodeling were determined in cancer progression (36). Among these, DNA methylation and nucleosome-mediated inflammatory gene expressions, aberrant histone methylation or acetylation-mediated glioma-associated macrophages/microglia (GAMs) polarization, and ncRNA-mediated high expression of inflammatory signaling pathways contribute to the transcription and growth of GBM tumor cells (37–40). These epigenetic modifications induce inflammatory cytokine release that accelerates the chronic inflammatory response in the TME of GBM, and reversing or inhibiting these changes are beneficial for longer survival in GBM patients, suggesting that epigenetic regulations of inflammation are important to GBM (10, 11). Therefore, we systemically reviewed the associated epigenetic changes and regulation in the inflammatory TME of GBM procession, aiming to comprehensively recognize epigenetic modifications of the inflammatory microenvironment in GBM.

## Inflammation Accelerates GBM Progression

Aberrant activation of inflammatory responses is a significant trait of GBM (41, 42), which endows GBM tumor cells with an immune evasion ability, thus causing immune tolerance of GBM to any therapies (38, 43). GBM tumor cells and GAMs are the main contributors to the inflammatory microenvironment (44, 45), exaggerating tumor invasion and relapse (46, 47). Moreover, GBM tumor cells also collaborate with the extracellular matrix, astrocytes, pericytes, and endothelial cells to secrete multiple inflammatory cytokines and chemokines that foster GAM infiltration and polarization (48–50), which forms a vicious cycle that accelerates the inflammatory response in the microenvironment and further aggravates GBM.

### GBM Tumor Cells Promote Inflammation

**Figure 1:**
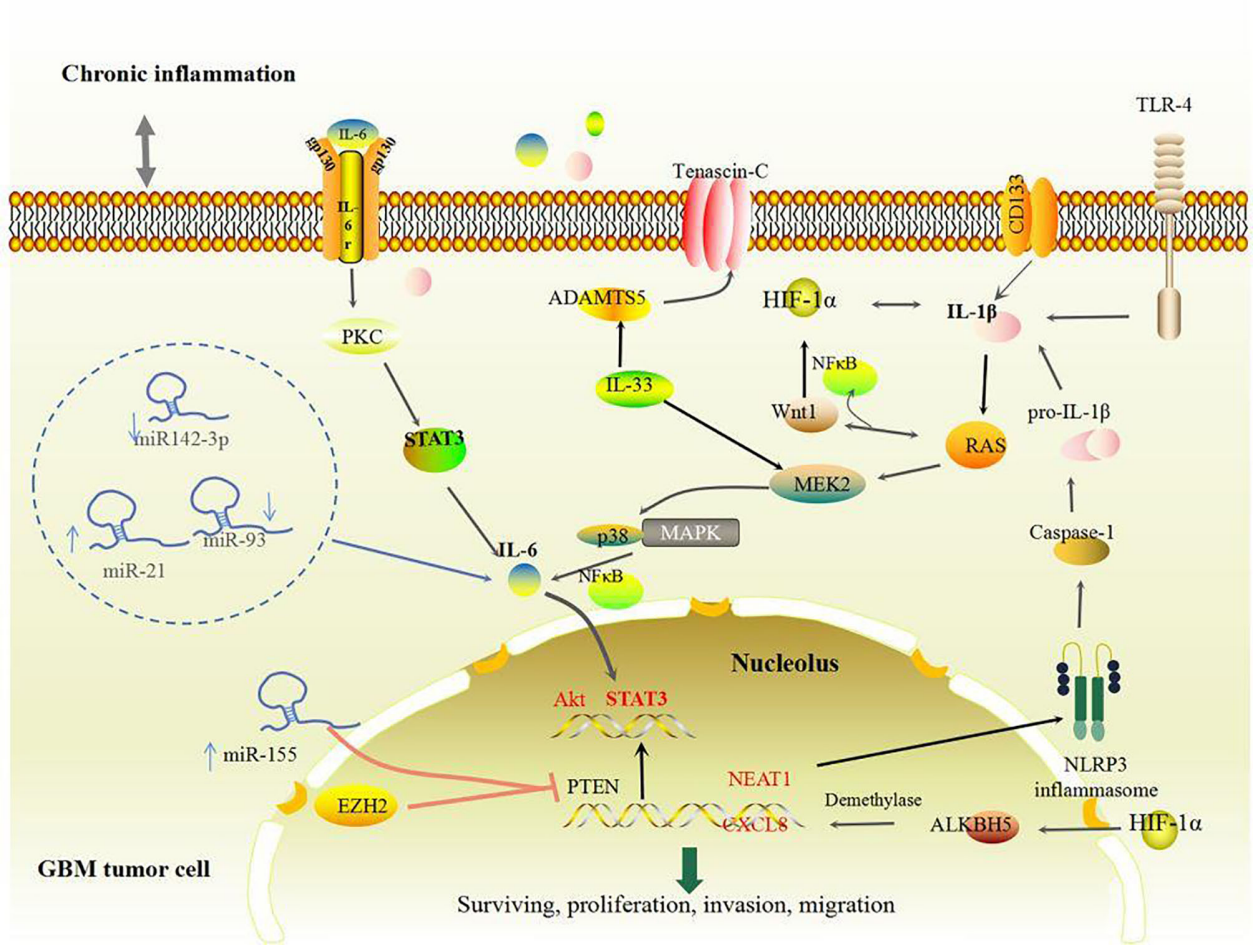
Inflammation-related changes in GBM tumor cells. GBM tumor cells produce abundant IL-1 and IL-6 through activating various pathways including STAT3, RAS, NFκB, NLRP3, and HIF-1α pathways, thus creating a chronic inflammatory environment, which benefits GBM growth.

GBM tumor cells exhibit a strong inflammatory signature that persistently produces pro-inflammatory cytokines at chronic low levels, thus generating a chronic inflammatory state in the TME (51). Represented by IL-1 and IL-6, primary mediators of GBM tumor cells in the inflammatory TME can orchestrate immunological activation and inflammatory signaling cascades such as hypoxia-inducible factor-1α (HIF-1α), Wnt-1, nuclear factor-kappa B (NF-κB), and STAT3 in GBM (52–55) (**Figure 1**). The members of the IL-1 subfamily including IL-1β and IL-33 drive tumor promotion and immune suppression (56). Activations of the NLRP3 inflammasome and CD133 in GBM tumor cells could induce the production of IL-1β and its downstream chemokines CCL3, CXCL3, and CXCL5 (57), which facilitate the migration, proliferation, self-renewal, and invasion of GBM (58–60). IL-1β activates both RAS and Wnt-1 which mediate the elevation of HIF-1α, thus inducing a HIF-1α/IL-1β autocrine loop in GBM tumor cells (61). Meanwhile, IL-1β also activates the p38 mitogen-activated protein kinase (MAPK)-activated protein kinase 2 (MK2)- human antigen R (HuR), toll-like receptor 4 (TLR-4), and other inflammation-associated signaling pathways that significantly increase the levels of IL-6 and IL-8 in GBM tumor cells, eventually developing an inflammatory TME in favor of GBM invasion and growth (61–63). Additionally, IL-33 induced by GBM tumor cells is another important inflammatory mediator that accelerates GBM proliferation, migration, and invasion (47, 64, 65). Interestingly, IL-33 could transform non-stem cells to GBM stem cells (66), activate disintegrin and metalloproteinase with thrombospondin motifs 5 (ADAMTS5) and EGFR by promoting the accumulation of tenascin-C (TNC), which in turn exacerbates GBM tumor cell proliferation (47, 64, 65, 67). Both subfamilies of IL-1 accelerate GBM relapse, however, blocking IL-1β and IL-33 can reduce tumor progression-associated compensation mechanisms including the Warburg effect (47, 68). Furthermore, emerging data suggest that GBM tumor cell-induced IL-6 displays a pro-tumorigenic role, and correlates with poor prognosis and GAM infiltration in GBM (67, 69, 70). IL-6 stimulates the invasion and growth of GBM tumor cells (71) by binding to heterogeneous membrane receptor complexes (formed by IL-6r and glycoprotein 130) and initiating typical IL-6 signal transduction (53), such as STAT3. Therefore, pro-inflammatory cytokines such as IL-1 members and IL-6 secreted from GBM tumor cells aggravate tumor-promoting inflammation and GBM growth.

### Glioma-Associated Microglia/Macrophages

**Figure 2:**
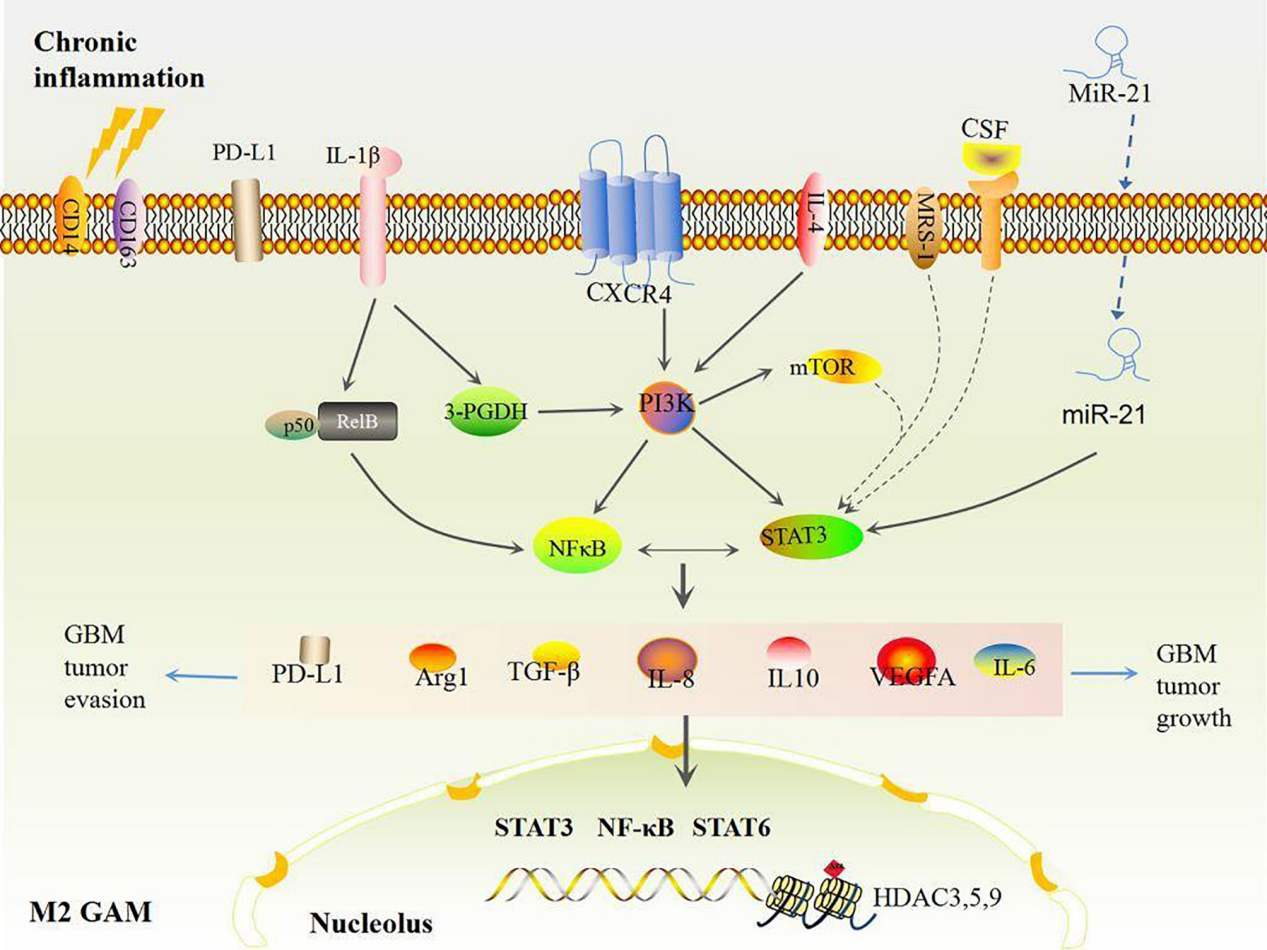
Chronic inflammation induced M2 GAMs. Under chronic inflammation, most GAMs are polarized to the M2 type by STAT3 and NFκB activation, which produces high levels of VEGF and immunosuppressive factors including IL-6, IL-8, IL-10, Arg1, TGF-β, and PD-L1, eventually inducing more M2 polarizations and accelerating GBM growth and evasion in turn.

GAMs are the domain members of immune cells in GBM when GSCs have a strong evasion ability (38). It is noteworthy that pro-inflammatory and angiogenic pathways including TNF, interferon (IFN), NF-κB, and hypoxia pathways in GAMs accelerate RAS-driven-GBM tumor cell proliferation *via* upregulation of IL-1β, VEGFA, CCL8, Arg1, CD274, and PD-L1 (72). High levels of inflammatory genes including *IL-6, IL-8, IL-1β, IL13RA1, IL13RA2, IL10RB, CXCR4, OSMR, CCR1, MDK, LIF, FAS, CCL2, CCL20, CXCL10, CXCL11*, and *CXCL14* as well as GAM makers such as CD14, CD163, TLRs, and CHI3L1 co-occur and are positively associated with poor survival in mesenchymal GBM (73). This suggests that GAM-associated inflammation is corrected with GBM progression. GAMs consist of two types of cells, GBM-associated macrophages and microglia. The former are preferentially recruited to the perivascular areas in early GBM, and GBM-associated microglia are localized to peritumoral regions (74, 75). Both types have polarized phenotypes with different functions: anti-tumorigenic and pro-tumorigenic phenotypes, which are defined as M1 or M2-like GAMs, respectively (50). Initially, stimulators like lipopolysaccharide (LPS), interferon (IFN)-γ, TNF, CD80, CD86, and IL-12A/B induce M1-like polarization by activating the TLR4-NF-κB and STAT1 signaling pathways (73, 76), whereas activations of *IL-4, IL-6, IL10, CD163, MSR1, MRC1, CD209, CLEC10A, CLEC7A, and CXCR4* induce M2-like polarization through the NF-κB/STAT3 pathway (73). In general, most GAMs display the M2 phenotype due to activation of STAT3 and NF-κB, secretions of immunosuppressive cytokines (e.g., IL-6, IL-10, IL-8, TGF-β1, MCP-1 and PGE-2), and colony stimulating factor (CSF) secretions of the TME in GBM (77–81). M2 GAMs further produce more Arg-1, TGF-β, IL-10, IL-6, and other abundant immunosuppressive cytokines, thus foster immunosuppression and pro-angiogenesis which contribute to growth and invasion of GBM after GBM tumor cells adapt to the pro-inflammatory TME (46). Additionally, other pathways including mTOR also increase activities of STAT3 or NF-κB in M2 GAMs that decrease immune reactivity and functional immune cell infiltration (80). Ultimately, these effects mediate GBM tumor immune evasion and growth (**Figure 2**), hence, reshaped GAMs have potential as a therapy mode in GBM.

### Cellular Communication in GBM

**Figure 3:**
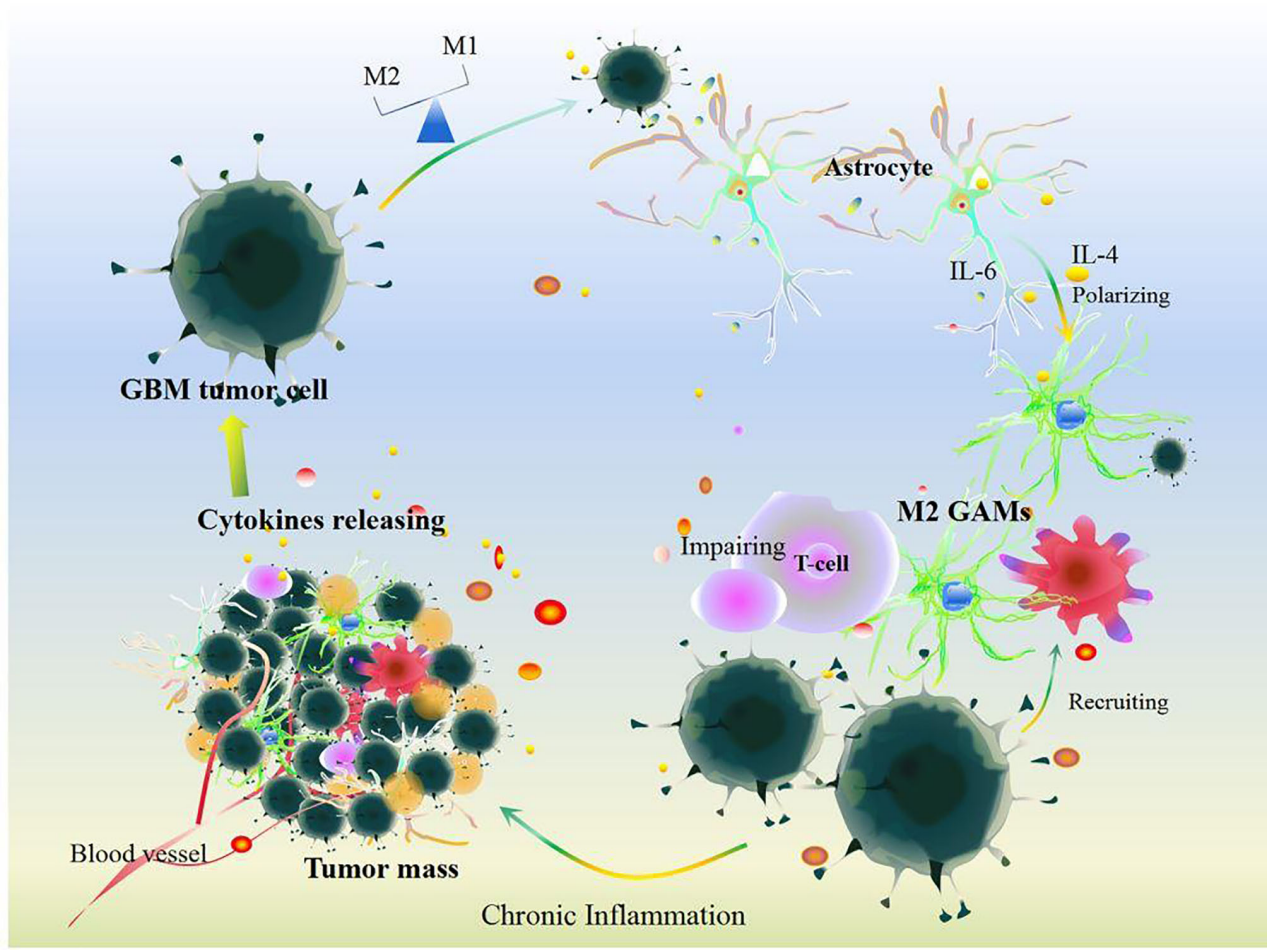
Cellular communication in GBM. Under chronic inflammation, cytokines released by cells including somatic cells and other immune cells can recruit GAM infiltration after the survey and chemotherapy and immunotherapy treatments in GBM. Additionally, GBM tumor cells induce GAM polarization resulting in disrupting the balance of M1 and M2 GAMs. Meanwhile, GBM tumor cells communication with astrocytes and other somatic cells could impair T cell function and release plenty of cytokines including IL-6, IL-4, IL-10, IL-8, IL-1, TGF-β, Arg-1, and VEGF. All these changes aggravate GBM tumor growth.

Cellular communication in GBM also participates in chronic inflammatory procession (**Figure 3**). At the primary state, the crosstalk between stromal cells and tumor cells that induces inflammatory cytokines like IL-2 can recruit neutrophils, mast cells, T cells, and B cells to the TME of GBM (44). In contrast, GBM tumor cells protect themselves by inducing IFN-γ, IL-10, IL-8, CCL2, IL-6, and TGFβ, which impair the anti-tumor ability of immune cells and reprogram immune cells to inflammatory phenotypes that produce inflammatory mediators, anagenetic factors, and PD-1/PD-L1, resulting in GBM tumor cell evasion and invasion (44, 82, 83). And then, GBM tumor cell-induced IL-6 activates STAT3 in astrocytes, which subsequently drives IL-6 to stimulate GBM tumor cells and further promotes STAT3 signals and enhances downstream events (84). Certainly, STAT3-dependent signal-mediated GBM tumor cells produce more IL-6 thus increasing the level of PD-L1 in immunosuppressive peripheral myeloid cells and facilitating tumor growth (85), which indicates that reciprocal activation of IL-6 and STAT3 continuously fosters chronic inflammation to exacerbate GBM growth and evasion (84). Interestingly, pro-inflammatory cytokines induce tumor granule neuron precursors differentiating into astrocytes (74), and then astrocyte-mediated IL-4 stimulates GAMs to produce insulin-like growth factor 1, thereby further promoting tumor progression (86). GBM tumor cells also foster pericytes and endothelial cells to produce more IL-10 and TGFβ by activating the IL-6/STAT3 signaling pathway, thus inciting tumor growth (87–89). Surrounding anti-inflammatory cytokines, including IL-8 and IL-10, shift M1 GAMs toward M2 GAMs (78), which would express Arg-1 (46) and contribute to abnormal angiogenic activity in GBM (46).

Furthermore, crosstalk between GBM tumor cells and GAMs is indispensable to the growth of GBM. Firstly, GBM tumor cell-induced IL-1β drives the HIF-1α-IL-1β autocrine loop to maintain a persistently elevated IL-1β level (61) that activates RelB/p50 complexes, thus attracting or polarizing GAMs (90). GBM tumor cells promote M2 GAM polarization by activation of STAT3, NF-κB, Wnt-3a, and mechanistic target of rapamycin (mTOR) (91), resulting in upregulation of IL-10 levels (91). And then, M2 GAMs also release IL-1β that activates protein kinase c (PKC)-delta and mediates phosphorylation of the glycolytic enzyme glycerol-3-phosphate dehydrogenase (3-PGDH), which subsequently activates phosphatidylinositol-3-kinase (PI3K) and promotes a feed-forward inflammatory loop in GBM tumor cells (68, 90). Moreover, GBM tumor cells together with GAMs generate a mass of succinate and lactate through the PI3K/HIF-1α pathway, thus aggravating local inflammation and GBM metastasis (27–31). Specially, lactate accelerates glycolysis, angiogenesis, and chronic inflammation by promoting IL-1β, IL-8, VEGF, and NF-κB signals, which ensures sufficient oxygen and nutrient supply for tumor cell proliferation (32). Collectively, GBM tumor cell communication with GAMs, immune cells, astrocytes, pericytes, and endothelial cells accelerates the development of a chronic inflammatory TME in GBM.

## Epigenetic Regulations of the Inflammatory Microenvironment in GBM

Epigenetic changes occur simultaneously with oncogenic events in cancer progression. In GBM, epigenetic modification including DNA methylation, histone modification, and non-coding RNA regulation could modulate the inflammatory genes-oncogenes loop and associated immunosuppression (92–95), providing a therapeutic strategy and comprehensively recognized pathogenesis of GBM by targeting the inflammatory TME (**Table 1**).

**Table 1:** Epigenetic control inflammatory response of GBM.

Target	Intervene method	Subjects	Results	Reference
EZH2	MC4040 and MC4041	Primary GBM tumor cells and U87 cells	Reduces H3K27me3 levels, impairs the pro-inflammatory phenotype of GBM by decreasing expressions of TGF-β, TNF-α, and IL-6, and restrains cell growth	(96)
*ALKBH5*	Knockdown	Raw264.7 cells, U87 and GL261 cells	Reverses m^6^A demethylation, decreases TAM recruitment, and inhibits *CXCL8* expression, reducing IL-8 secretion and restraining cell growth	(24)
*IL-8*	Knockdown	U251 human glioma cell lines, patient-derived xenograft glioma specimens, athymic nude mice	Reduction of IL-8 abolishes methylation of the H3K27 and H3K9 residues, restraining cell growth	(97)
*IL-8* promoter	Prostaglandin E2, 5-aza-2’-deoxycytidine and HDAC inhibitors	Human 1321N1 (derived from grade II astrocytoma) and A-172 cell lines (derived from grade IV glioblastoma)	Reverses methylation status of IL-8 prompter, restrains cell growth	(98)
*IL-6;*	Knockdown of DNMT1; IL-6 neutralization; miRNA 142-3p mimics	NBE-cultured cell lines, CSC-like GBM tumor cells	Inhibits tumorigenicity and blocks *IL-6*, *HMGA2*, and *SOX2* expression in GBM	(34)
*p38a* *MAPK*	RNAi p38a MAPK knockdown	U251 human GBM tumor cells	Reduces IL-6 secretion and suppresses U251 GBM cell migration and invasion in the presence of inflammatory cytokines	(99)
lncRNA *SNHG15*	Palbociclib	GBM tumor cells, human microglial cell line (HMC3)	Regulates the lncRNA *SNHG15*/CDK6/*miRNA-627* circuit and reduces polarization of M2 GAMs, decreasing GBM tumorigenesis and increasing temozolomide sensitivity	(100)
HDAC	Valproic acid and sodium butyrate	Microglia	Leads to a decrease of the inflammation response of microglia	(101)
HADC 5/9	Trichostatin A or valproic acid	Glioma-polarized microglia	HDAC inhibitors block acquisition of transcriptional memory in glioma-polarized microglia	(37)
HDAC1/2	Panobinostat, vorinostat, and romidepsin	GBM-microglia	Reduces glycolysis in a c-Myc-dependent manner and lowers ATP levels	(102)
*miR-21*	Konckdown/pacritinib	GBM-GAMs	Reduces the polarization of M2 GAMs and levels of VEGF, TGF-β1, and IL-6 by decreasing expression of *Sox2, PDCD4*, and *STAT3*, which inhibits GBM tumorigenesis	(103, 104)
*miR-124*	*miR-124*-loaded extracellular vesicles	GBM-microglia	Inhibits M2 GAM polarization and suppresses tumors by modulating STAT3 activity and recruiting natural killer cells to the TME	(105)

### DNA Methylation

DNA methylation is an epigenetic modification of DNA that is important for the normal regulation of transcription, embryonic development, genomic imprinting, genome stability, and chromatin structure, and is controlled by DNA methyltransferases, methyl-CpG binding proteins, and other chromatin-remodeling factors, thereby controlling gene expression (36). DNA methylation loss of oncogenes can activate inflammatory oncogene expression and extensively promote GBM growth (106, 107). GBM patients with an unmethylated O6-methylguanine-DNA methyltransferase (*MGMT*) promoter significantly show an elevated expression of inflammatory genes such as *IL-6, CCL2, CCXCL2, HLA-A, and Serum amyloid A1* (108, 109), similar to methylation of *MGMT*, positively associated with IL-6-mediated primary GBM procession (110). This implies that inflammation is associated with high expression of *MGMT*. Additionally, *N6*-methyladenine (*N* ^6^-mA) is highly enriched in histone 3 lysine 9 trimethyl (H3K9me3) in GBM tumor cells, which is induced by ALKBH and exacerbates GSCs’ self-renewal and proliferation (111, 112). Specially, the demethylase activity of ALKBH5 has been demonstrated as an indispensable role in regulating the conditions of hypoxia and inflammatory TME in GBM and is required for NLRP3 inflammasome-related *CXCL8* and *NEAT1* gene activation (24, 113, 114). Silencing *MGMT* or *ALKBH5* of GBM tumor cells could inhibit GAM infiltration and repress *NEAT1* and *CXCL8* expression, eventually suppressing GBM tumor cell growth and lengthening the survival of GBM patients (24, 113, 115). This suggests that regulation of the DNA methylation state of inflammation-associated genes is beneficial for GBM treatment.

### Histone Modification

Histone modification is covalent post-translational modification to histone proteins including methylation, phosphorylation, acetylation, ubiquitylation, and sumoylation, and regulates gene expression by altering chromatin structure or recruiting histone modifiers (116). Histone methylation and acetylation are found frequently in GBM. On one hand, enhancer of zeste homologue 2 (EZH2) and lysine 27 on histone H3 (H3K27) methyltransferase (H3K27me) highly mutate in GBM tumor cells, and lead to a poor outcome of GBM (96). EZH2 increases H3K27me3 in the promoter of *PTEN* thus silencing *PTEN* and activating the threonine protein kinase B (AKT)/mTOR pathway, promoting GBM tumor cell proliferation and metastasis (117). Interestingly, EZH2 inhibitors (MC4040 and MC4041) not only reverse the pro-inflammatory phenotype of GBM U-87 and GL-1 cells by downregulating the levels of H3K27me3, but also inhibit epithelial-mesenchymal transition and migration of primary GBM tumor cells by reducing the level of VEGF and VEGFR1, resulting in inhibiting GBM cell proliferation and invasion of GBM (96). On the other hand, histone deacetylases (HDACs) are also positively associated with poor clinical features of GBM (118, 119). HDAC activity is greatly increased in GBM by activating STAT6, IFR-3, IFR-4, NFκB, TLR, and IFN (120), and is regarded as the main effector of epigenetic alterations in M2 GAMs (121, 122). During the GBM process, hyperactivity of HDAC3, 5, and 9 is expressed in IL-4-induced M2 GAMs (101, 123) that increase the levels of TGFβ and IL-10, resulting in exacerbating the immunosuppressive capacity of GBM tumor cells (124). It is worth noting that HDAC inhibitors can thwart M2-type polarization of GAM and retard GBM tumor growth (37, 125) by restricting activation of histone marks or targeting the STAT6 signaling pathway in a HDAC3-dependent manner (123, 124, 126). Unfortunately, EZH or HDAC suppression also induces M1 GAMs secreting IL-1β and IL-6, and activates STAT3 signals in GBM tumor cells, leading to an aggravated inflammatory response in the TME of GBM (124, 127). Thus, other histone modifications and more effective histone-regulating methods of inflammation in GBM need further exploration.

### Non-Coding RNA Modification

Non-coding RNAs (ncRNAs) related to epigenetic regulation consist of two main groups: long ncRNAs (lncRNAs) and short ncRNAs including miRNA, siRNA, intronic RNA, repetitive RNA, piRNAs, snoRNAs, and lincRNAs, which generally act as cis-acting silencers, but also as trans-acting regulators of site-specific modification and imprinted gene-silencing (128, 129). Among these, miRNA has been frequently explored in GBM progression and invasion (130). A variety of miRNAs, including *miR-21*, *miR-26*, *miR-221/222*, *miR-210*, *miR-155*, and *miR-10b*, promote GBM cell proliferation, apoptosis, and growth by targeting *PTEN* expression and active *Akt* and *HIF3α*. Recently, a growing number of studies revealed the relationship between miRNAs and inflammation (100, 105, 131–133). The *MiR142-3p* promoter is methylated which decreases *miR142-3p* gene expression and increases the level of IL-6, thus promoting GBM invasion and migration in a DNA methyltransferase (DNMT) 1-dependent manner (34). In addition, inhibition of *miR-93* in GBM tumor cells fosters an inflammatory microenvironment *via* increasing the levels of IL-6, IL-8, IL-1β, granulocyte-colony stimulating factor, leukemia inhibitory factor, COX2, and CXCL5 (131). *MiR-155* induced by inflammatory cytokines, such as IL-1β and TNFα, mediates mesenchymal transition of GBM tumor cells and GBM growth (134, 135). Nevertheless, inhibition of *miR-155* restrains the proliferation, migration, and invasion of GBM tumor cell growth by activation of STAT3 (132, 133). Interestingly, *miR-124*-loaded extracellular vesicles suppress GBM tumors by activating STAT3 activity and recruiting natural killer cells to the TME (100, 105). Downregulation of *miRNA-125b* or inhibition of mitogen-activated protein kinase (MAPK) mRNA could restrain the p38/MAPK pathway and reduce IL-6 secretion, which suppresses GBM tumor cell migration and invasion (99, 130, 136). *MiR142-3p* mimics could block *IL-6, HMGA2*, and *SOX2* expression in GBM tumor cells that inhibit tumorigenicity (34). Besides, upregulation of *miR-93* can significantly suppress proliferation, migration, and angiogenesis of GBM tumor cells by alleviating the inflammatory environment (131). *MiR-21* in exosomes or extracellular vesicles induced by GBM tumor cells can be incepted by GAMs and upregulate IL-6, TGF-β, and Arg-1 in M2 GAMs (103, 104), resulting in downregulating the immune response and accelerating growth and invasion of GBM tumor cells (103). However, inhibition of *miR-21* significantly reduces the polarization of M2 GAMs and decreases levels of VEGF, TGF-β1, and IL-6 by decreasing activities of *Sox2, PDCD4*, and *STAT3*, ultimately inhibiting GBM tumor cell growth (104). Collectively, RNA interference is important to GBM treatment through multiple regulations of inflammation-related signals.

## Conclusions and Perspectives

The inflammatory TME as the primary contributor to the pathogenesis of GBM, which consists of various functional cells including GBM tumor cells, GAMs, and other non-tumor cells, and the autocrine and reciprocal paracrine of these above cells form a chronic low-grade inflammation state that benefits proliferation, invasion, migration, and evasion of GBM. While, epigenetic modification is a new perspective for understanding the pathogenesis of GBM. Here, we find that epigenetic regulators including DNMT, EHZ, HDAC, and miRNAs are involved in the inflammation of GBM, reminding us that epigenetic control of the inflammatory TME may be a rewarding therapy for GBM treatment. However, some problems still need to be further explored in the future. Firstly, the different epigenetic changes between primary and secondary GBM need to be explained, which is meaningful to identify and diagnose the two subtypes. Secondly, DNA methylation and histone modification including phosphorylation, ubiquitylation, and sumoylation of the inflammatory TME in GBM need elaborating. And then, the relationships of the inflammatory TME between other epigenetic modifications, such as chromosome structure, nucleosome transcription, long ncRNAs, and other short ncRNAs need comprehensively uncovering. Finally, the interactive influences of different epigenetic modifications in GBM need to be explained. Overall, we provide insight into the epigenetic regulation of inflammation in GBM and reveal the relationship between inflammation and GBM progression, suggesting some future directions of GBM underlying epigenetic modification.

## Author Contributions

NC drafted the manuscript and drew the figures. CP and DL conceived and revised the review. All authors contributed to the article and approved the submitted version.

## Funding

Financial support by the National Natural Science Foundation of China (82104477 and U19A2010) and multidisciplinary interdisciplinary innovation team for multidimensional evaluation of southwestern characteristic Chinese medicine resources (NO. ZYYCXTD-D-202209), and special support from the China Postdoctoral Science Foundation (2019M663456 and 2019TQ0044), Xinglin Scholar Research Promotion Project of Chengdu University of TCM (BSH2019008) are gratefully acknowledged.

## Conflict of Interest

The authors declare that the research was conducted in the absence of any commercial or financial relationships that could be construed as a potential conflict of interest.

## Publisher’s Note

All claims expressed in this article are solely those of the authors and do not necessarily represent those of their affiliated organizations, or those of the publisher, the editors and the reviewers. Any product that may be evaluated in this article, or claim that may be made by its manufacturer, is not guaranteed or endorsed by the publisher.
